# RNA-seq analyses of the midgut from blood- and serum-fed *Ixodes ricinus* ticks

**DOI:** 10.1038/srep36695

**Published:** 2016-11-08

**Authors:** Jan Perner, Jan Provazník, Jana Schrenková, Veronika Urbanová, José M. C. Ribeiro, Petr Kopáček

**Affiliations:** 1 Institute of Parasitology, Biology Centre of the Czech Academy of Sciences, Branišovská 31, 370 05, České Budějovice, Czech Republic; 2 Institute of Entomology, Biology Centre of the Czech Academy of Sciences, Branišovská 31, 370 05, České Budějovice, Czech Republic; 3 Section of Vector Biology, Laboratory of Malaria and Vector Research, National Institute of Allergy and Infectious Diseases, National Institutes of Health, Bethesda, Maryland, United States of America

## Abstract

Adult females of the genus *Ixodes* imbibe blood meals exceeding about 100 times their own weight within 7‒9 days. During this period, ticks internalise components of host blood by endocytic digest cells that line the tick midgut epithelium. Using RNA-seq, we aimed to characterise the midgut transcriptome composition in adult *Ixodes ricinus* females during early and late phase of engorgement. To address specific adaptations to the haemoglobin-rich diet, we compared the midgut transcriptomes of genetically homogenous female siblings fed either bovine blood or haemoglobin-depleted serum. We noted that tick gut transcriptomes are subject to substantial temporal-dependent expression changes between day 3 and day 8 of feeding. In contrast, the number of transcripts significantly affected by the presence or absence of host red blood cells was low. Transcripts relevant to the processes associated with blood-meal digestion were analysed and involvement of selected encoded proteins in the tick midgut physiology discussed. A total of 7215 novel sequences from *I. ricinus* were deposited in public databases as an additional outcome of this study. Our results broaden the current knowledge of tick digestive system and may lead to the discovery of potential molecular targets for efficient tick control.

Ticks acquired the habit of blood feeding more than 100 million years ago and are the main vectors for pathogens of humans and livestock globally^1^,^2^. Unlike blood-feeding mosquitoes, all tick life stages feed exclusively on host blood; adult *Ixodes* spp. females feed on their hosts for 7‒9 days. As tick feeding progresses, tick digest cells develop along the tick gut epithelium^3^, where nutrient endocytosis and lysosome maturation facilitate intracellular digestion^4^. Extensive characterisations of tick midguts have been conducted in various tick species, at both transcript^5^,^6^,^7^,^8^,^9^ and protein^6^,^9^ levels, using massive parallel sequencing and mass spectrometry, respectively. All these studies have been carried out using pooled samples of midgut preparations dissected from a number of ticks fed naturally on laboratory animals. This approach, however, does not reveal expression of novel transcripts induced by blood meal components.

Using an artificial feeding system implemented for the European Lyme disease vector *Ixodes ricinus*^10^, we have recently demonstrated that ticks fed on red blood cell (RBC)-depleted serum can successfully engorge and lay eggs. These eggs, however, were sterile and unable to give rise to viable progeny, indicating the ultimate dependence of ticks on haem acquisition from host haemoglobin to sustain embryonic viability^11^. We hypothesised that blood meal depletion of RBCs (serum feeding) might reveal adaptive traits that have enabled ticks to digest haemoglobin intracellularly, with concomitant haem acquisition and transport to the haemocoel, supplying haem metabolic demands in peripheral tissues or detoxification of haem excesses via transport to haemosomes^12^. To reveal if adult female ticks express specific transcripts associated with blood meal processing in response to dietary haemoglobin we have subjected gut RNA extracts from individual blood- and serum-fed ticks to Illumina RNA sequencing (RNA-seq) and have compared their assembled transcriptomes.

To characterise the two important phases of blood-meal uptake and digestion^3^, namely the slow phase (feeding initiation) and rapid phase (preceding tick detachment from the host) of engorgement, we have isolated RNA from the midgut of ticks fed for 3 days and 8 days. The midgut transcriptomes from single *I. ricinus* females revealed substantial temporal differences in gene expression between these two phases. However, the number of genes whose expression was affected by the presence/absence of haemoglobin in the diet was surprisingly low. These findings may help to better understand the physiological processes that are indeed crucial for tick feeding and reproduction.

## Results and Discussion

### Sample preparation and RNA-seq design

**Figure 1:**
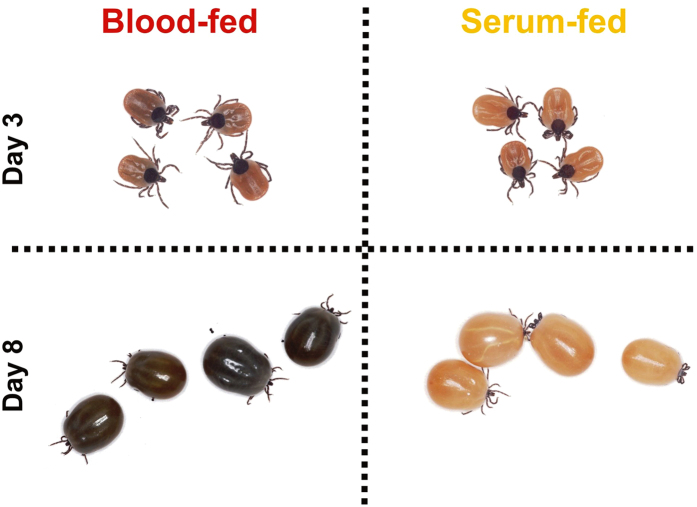
Blood- and serum-fed adult *Ixodes ricinus* females used in this study. First-generation siblings *I. ricinus* females were membrane-fed for 3 days (partial engorgement) or 8 days (full engorgement) with either reconstituted bovine blood or bovine serum. At particular time points, ticks were dissected and individual midgut caeca were used for RNA extractions. Resulting RNA extracts from individual ticks were used for RNA-seq analyses.

**Figure 2:**
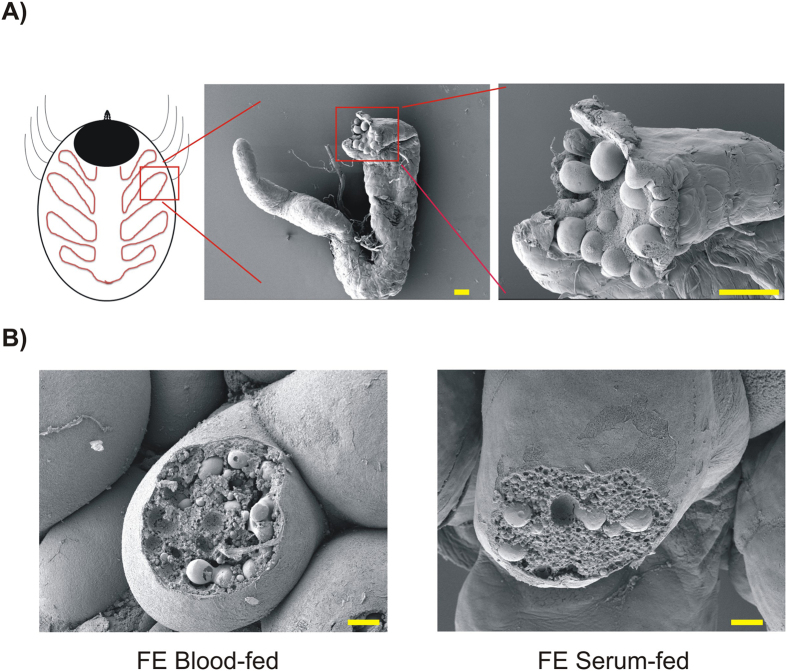
Scanning electron microscopy of tick gut caecum and digest cells. **(A)** Illustration of tick gut caecum dissected from a partially-fed adult *I. ricinus* female. Such caeca were used for RNA-seq analyses. Scale bars indicate 100 μm. **(B)** Manually disrupted digest cells maturing along tick midgut epithelium from blood- (left) and serum-fed (right) fully engorged adult *I. ricinus* females. Note that digest cells from either tick contain both small and large digestive vesicles. Scale bars indicate 10 μm.

We have recently demonstrated, using artificial membrane feeding^10^, that ticks require dietary haemoglobin as their ultimate source of haem since they are not capable of haem biosynthesis^11^. Apart from the fact that feeding ticks on haemoglobin-depleted serum led to aborted embryogenesis, no other obvious physiological effect was observed during the process of tick feeding and oviposition. Using RNA-seq analysis, we have examined transcriptomic changes in the adult tick gut in response to blood-feeding (BF) and serum-feeding (SF) in a temporal-dependent manner. In order to increase the consistency and integrity of RNA-seq data and minimise individual-specific deviations in expression among tick females, we have raised, under laboratory conditions, a cohort of genetically related adult *I. ricinus* siblings (first generation sisters). Ticks were dissected at two time points: day 3 of feeding (3D), which corresponds to the to the slow-feeding phase and day 8, representing fully engorged females (FE)^3^,^13^. Four females were dissected per time point and per diet (Fig. 1) with each female being represented by a single cDNA library (in total, 16 libraries were prepared). For library preparation, only females with similar weights were selected (Supplementary Figure S1). A catalogue of individual females selected for library preparations was prepared and library names were allocated (Supplementary Figure S1). RNA extractions were performed from single midgut caeca comprising developed digest cells containing both small and large digestive vesicles^14^ from both BF and SF ticks (Fig. 2).

### Tick gut transcriptome re-assembly and mapping of reads

*De novo* assembly of the *I. ricinus* midgut transcriptome was recently performed for the early stage of adult female feeding (up to 36 hours after attachment)^7^. Our libraries were sequenced using a MiSeq protocol yielding 300 nt transcripts that aided re-assembly of longer transcripts^7^,^15^. From MiSeq sequencing, nearly 3 million reads per library, averaging 280 bp in length, were obtained. HiSeq sequencing yielded an average of 13 million single-end reads per library, averaging 120 bp in length. A summary of the reads, after removal of Illumina primers and trimming low quality base (smaller than 20) values, is provided in the Supplementary Information (Supplementary Tables S1 and S2) for MiSeq and HiSeq protocols, respectively. HiSeq reads were then mapped onto our midgut transcriptome re-assembly. The coding sequences were deposited in DDBJ/ENA/GenBank under accession number GEFM00000000 as a Transcriptome Shotgun Assembly project; BioProject: PRJNA311553. The generated contigs, their respective expression values, and putative protein characteristics are presented in a hyperlinked Excel spreadsheet (further referred to as Source data 1) so that comparisons of contig levels can be made between individual ticks. Sequences were deposited on the NIH exon server and are available at: http://exon.niaid.nih.gov/transcriptome/Ixric-MG/Ir-web.xlsx.

### Tick gut transcriptome composition in response to time or diet

**Figure 3:**
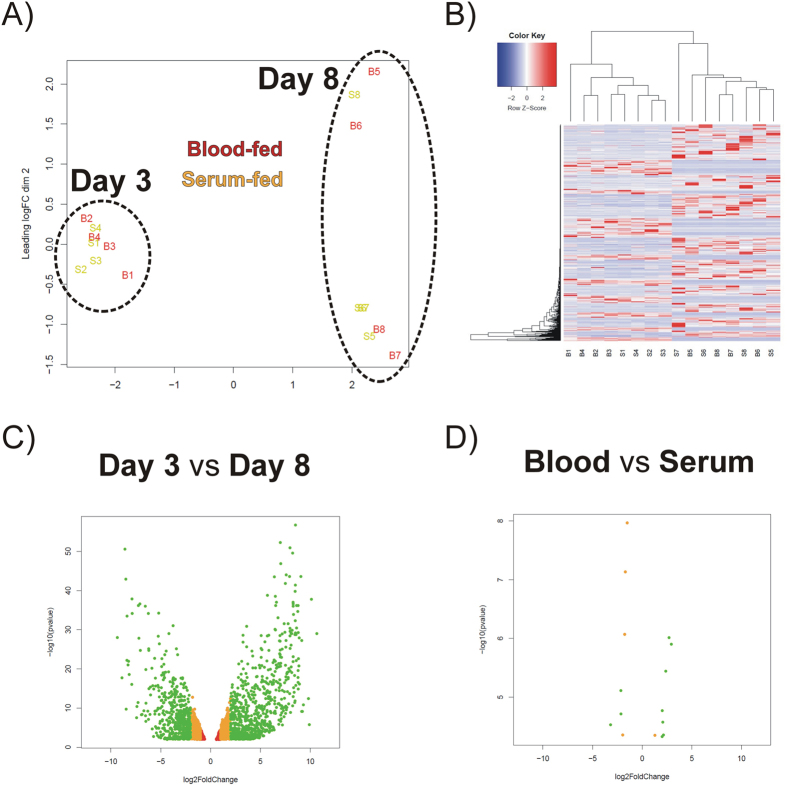
Transcriptome analysis. The transcriptomes from individual blood-fed (B1‒B8) and serum-fed (S1‒S8) ticks were characterised using an EdgeR package. **(A)** MDS plot of 16 libraries indicating the related nature of transcriptomes from a given time-point of feeding. **(B)** Heat map of differentially expressed intestinal contigs in individual libraries. The gene expression (RPKM) values for each gene were normalised to the standard normal distribution in order to generate Z-scores. **(C)** Volcano plot visualising the number of differentially expressed genes (2676) with respect to tick feeding stage. **(D)** Volcano plot visualising the number of differentially expressed genes (15) with respect to tick RBCs (+/−) diet composition.

To visualise similarities between individual libraries, a multidimensional scaling (MDS) plot was generated. Visualisation indicates that tick gut transcriptomes are subject to distinct temporal-dependent changes (Fig. 3A). The generated heat map shows that differentially expressed gene transcripts derived from the gut of Day 3 ticks cluster well together, while individual transcripts derived from the gut of FE ticks cluster amongst themselves (Fig. 3B). EdgeR analysis, using a set of 13,437 transcripts (RPKM ≥ 1 in at least one library), identified 2,676 transcripts that were differentially expressed when the time variable was considered (using an FDR of 0.05 or smaller) (Fig. 3C), while only 15 transcripts were found to be differentially expressed (using an FDR of 0.05 or smaller) (Fig. 3D) when the variable diet was considered. We have listed the most up-regulated contigs (RPKM ≥ 3) in the midgut at day 3 or day 8 of feeding in Tables 1 and 2, respectively. Some of the “early gut transcripts” encode enzymes generally associated with the physiology of tick salivary glands, such as phospholipase A2 or glycine-rich secreted cement protein. To confirm our observation, we have generated tissue-specific cDNA sets from both Day 3 and Day 8 ticks to test tissue-specific expression. We have confirmed that most of the listed up-regulated transcripts displayed a gut-specific expression pattern. Only contigs IrSigP-110295 and IrSigP-109251 were shown to be predominantly expressed in the fat body-trachea-associated complex of Day 3 ticks (Supplementary Figure S2). To further confirm that expression of these genes is linked to the slow feeding phase of engorgement, we have run an RT-qPCR analysis of cDNA samples prepared from the tick midgut during and after feeding. We confirmed that all genes were expressed only during the slow feeding phase (Supplementary Figure S2). Most of the contigs that were up-regulated in Day 8 ticks were gut-specific, except for contigs Ir-121498, Ir-113115, Ir-97790 and Ir-113452 (Supplementary Figure S3). The tissue profile of contig IrSigP-108669, encoding vitellogenin 2, displays gut and trachea-fat body complex-specific expression^11^. To further confirm that expression of these genes was linked to the rapid phase of engorgement, we performed an RT-qPCR analysis of cDNA samples prepared from the tick midgut during and after feeding. We confirmed that all of these genes were expressed only during the rapid engorgement phase and after tick drop-off (Supplementary Figure S3). We have also identified the most abundant contigs overall that are listed in the Source data 1 (columns GA‒GP).

### Transcriptomic changes in response to blood meal

**Figure 4:**
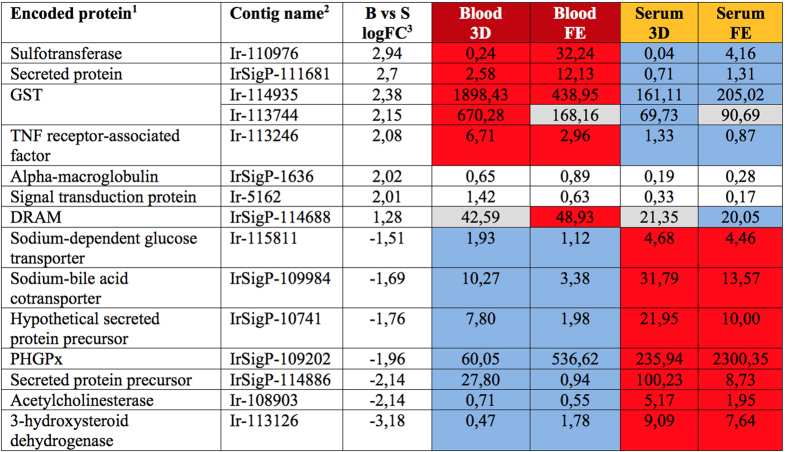
Overview of average RKPM values for contigs differentially expressed between serum- and blood-fed ticks. Encoded proteins and their respective average RPKM values in particular libraries are listed. ^1^Abbreviations: GST-glutathione S-transferase; TNF-tumor necrosis factor; DRAM-damage-regulated autophagy modulator; PHGPx-Phospholipid hydroperoxide glutathione peroxidase; ^2^For contig names, sequences and other and RKPM data, see the Source data 1 at http://exon.niaid.nih.gov/transcriptome/Ixric-MG/Ir-web.xlsx; ^3^BvsS logFC represents the base 2 logarithm of the fold change in transcript expression when the Blood treatment was compared with the Serum treatment. 3D, FE-indicate time periods of feeding 3 days and 8 days (fully engorged), respectively. Background color coding: No colour-low expression; Grey colour-insignificant differences; Red vs. blue-significant differences.

**Figure 5:**
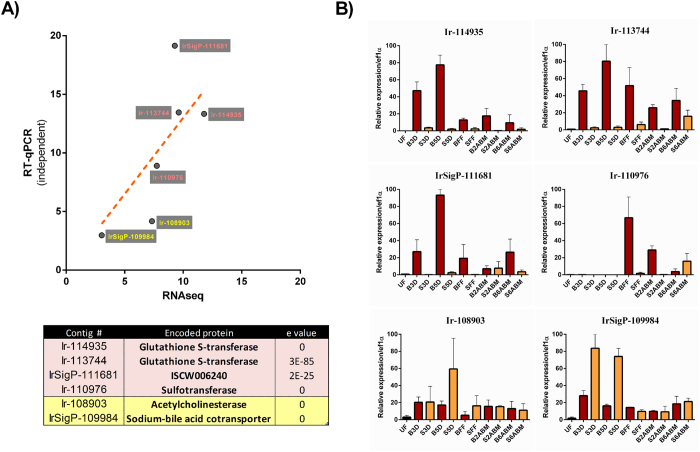
Biological (independent) RT-qPCR validation of RNA-seq data. **(A)** Correlation between fold change (up-regulation) of RT-qPCR (independent cDNA sets from unrelated ticks collected in the wild) and RNA-seq data on selected transcripts. The table of selected differentially expressed genes for blood-fed ticks and serum-fed ticks is shown below the graph. **(B)** RT-qPCR analyses of differentially expressed genes in the tick midgut dissected from *I. ricinus* females during and after feeding. Data were obtained from three independent cDNA sets, and normalised to *elongation factor 1 (ef1α*). UF-unfed; B-blood-fed; S-serum-fed; 3D, 5D, FF-indicate time periods of feeding 3 days, 5 days, 8 days (fully fed), respectively; 2 ABM and 6 ABM indicate time periods after blood meal, 2 days and 6 days, respectively. Mean and SEM are shown, n = 3 (biological replicates).

Although the gut transcriptomes showed substantial feeding phase dependent changes, only few transcripts displayed differential expression in response to dietary RBCs (+/−). The fifteen transcripts with statistically significantly different expressions between BF and SF ticks are shown in Fig. 4. Four of the differentially expressed genes encode enzymes participating in antioxidant or detoxification networks, namely two paralogues of glutathione S-transferase (GST), one homologue of sulfotransferase (SULT), and one homologue of phospholipid hydroperoxide glutathione peroxidase. Three of the differentially expressed genes encode membrane proteins: a sodium-bile acid cotransporter, a sodium-dependent glucose transporter, and DNA damage-regulated autophagy modulator protein (DRAM). The remaining differentially expressed genes encode acetylcholinesterase, alpha-macroglobulin, 3-hydroxysteroid dehydrogenase, and unknown proteins IrSigP-111681, IrSigP-10741, and IrSigP-114886. To validate our RNA-seq data, a direct RT-qPCR analysis was carried out using RNA samples used for library construction and this confirmed the differential expression of selected contigs, although the RT-qPCR analysis tended to slightly overestimate differences in transcript levels (Supplementary Figure S4). To verify the biological validity of the differentially expressed genes, we have performed RT-qPCR of selected contigs on cDNA originating from unrelated ticks collected in the wild and fed with different batch of reconstituted bovine blood or serum, using the membrane feeding system. We have plotted their mean up-regulation values obtained from RT-qPCR against those obtained from RNA-seq (Fig. 5A). To extend the time-points, we inspected their expression profiles throughout the entire blood- or serum-feeding period and up to six days after detachment (Fig. 5B). We determined that both GST encoding contigs (Ir-114935 and Ir-113744) and contig IrSigP-111681 were consistently up-regulated throughout blood-feeding. A SULT-encoding contig (Ir-110976) was up-regulated by blood-feeding at FE and 2 days after blood meal (2 ABM). Acetylcholinesterase-encoding contig (Ir-108903) did not show any significant up-regulation during serum-feeding, while the sodium-bile acid co-transporter-encoding contig (IrSigP-109984) was up-regulated at days 3 and 5 of serum feeding (Fig. 5B). To reveal whether diet composition induced expression changes in gut-specific or more systemically expressed genes, we carried out an RT-qPCR analysis of selected differentially expressed genes in various tick tissues. While GSTs and SULT displayed gut-specific expression, contigs encoding an unknown protein, the sodium-bile acid cotransporter, and acetylcholinesterase displayed more systemic expression (Supplementary Figure S5). Below, we briefly discuss the known homologues of those differentially expressed genes affected by a blood or serum diet.

Glutathione S-transferase (GSTs) was shown to be up-regulated in blood-fed ticks. GSTs, formerly called ligandins, are notorious for showing affinities to a broad array of ligands. Several classes of GSTs were reported to have high affinities to haem-like compounds^16^. Interestingly, levels of GST contigs were shown to be up-regulated in a comparative analysis of *C. elegans* fed on medium containing differing levels of supplemented haemin^17^. Here, we have shown that two contigs (Ir-114935 and Ir-113744) were up-regulated upon blood-feeding. BLAST analysis of these sequences against the available *I. scapularis* genome^18^ revealed that Ir-113744 is most likely an intron-containing version of Ir-114935, which might have originated from pre-mRNA co-isolation.

Sulfotransferase (Ir-110976) was shown to be up-regulated by blood-feeding in Day 8 ticks. These enzymes catalyse the transfer of sulfonic groups to a broad range of substrates. The resulting molecules become more soluble in the cellular environment and are easier to metabolise/detoxify^19^. It is tempting to speculate that the encoded sulfotransferase may have an affinity towards haem in the tick digest cells and may facilitate its dispatch/detoxification. Further work is needed to explore the affinity of sulfotransferase towards an array of substrates in order to obtain a better insight into its biological function.

DNA damage-regulated autophagy modulator protein (IrSigP-114688; DRAM) was up-regulated in blood-fed ticks at both time points, Day 3 and Day 8. The encoded protein has an estimated molecular weight of 27 kDa and 6 predicted membrane helices (Source data 1, Column N and S, respectively). A related molecule was reported to display stress-responsive transcriptional suppression in human cells^20^. This result indicates that the serum-fed ticks may be subject to enhanced oxidative stress in their intestinal digest cells. A similar hypothesis proposed that sugar-fed mosquitoes, in comparison to blood-fed mosquitoes, experience enhanced oxidative challenge in their midgut^21^.

Acetylcholinesterase (Ir-108903), in contrast, was up-regulated in serum-fed ticks. The encoded enzyme suggests a possible involvement in neurological perception of received diets. Detailed screening of acetylcholinesterase inhibitors may be a fruitful strategy towards formulation and exploitation of anti-tick intervention initiatives. Such screening was recently performed against acetylcholinesterase from the herbivorous mite *Tetranychus cinnabarinus*^22^.

Sodium-dependent glucose transporter (Ir-115811) was up-regulated in Day 3 and Day 8 serum-fed females. The encoded protein, with an estimated molecular weight of 50 kDa and 12 predicted membrane helices (Source data 1, Column N and S, respectively), belongs to families of major facilitators. Members of these families in herbivorous mites were reported to show significant changes in their transcriptional responses to xenobiotics^23^,^24^.

Phospholipid hydroperoxide glutathione peroxidase (IrSigP-109202) was shown to be up-regulated in serum-fed Day 8 ticks. These enzymes are engaged in decomposing lipid hydroperoxides. It was reported that protein levels and enzymatic activity of phospholipid hydroperoxide glutathione peroxidase from *Schistosoma japonicum* can be stimulated by incubation of adult worms with millimolar amounts of oxidative molecules, such as paraquat or hydrogen peroxide^25^.

Sodium-bile acid cotransporter (IrSigP-109984) was shown to be up-regulated in Day 8 serum-fed ticks. The encoded ~50 kDa protein contains 9 predicted membrane helices (Source data 1, Column N and S, respectively). The sodium-bile acid cotransporter is a member of the solute carrier protein family 10 (SLC 10) that comprises influx transporters of bile acids, steroidal hormones, various drugs, and several other substrates^26^.

### Gene ontology analysis

**Figure 6:**
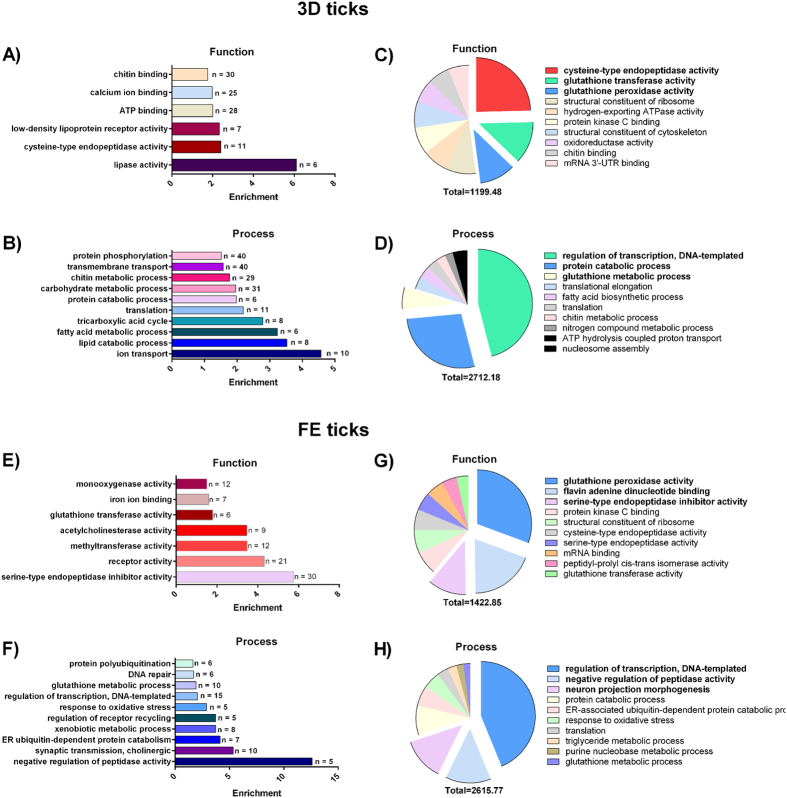
Gene ontology analysis. Bar graphs show the enriched GO terms of molecular function **(A,E)** and biological process **(B,F)** for 3D ticks **(A,B)** and FE ticks **(E,F)**; n indicates the number of contigs concerned. Results are shown only for E value match of <1e-15 and n ≥5. Pie charts show absolute representation of GO terms of molecular function **(C,G)** and biological process **(D,H)** for 3D ticks **(C,D)** and FE ticks **(G,H)**; sum of RPKM values are shown below the charts. The three most represented categories are highlighted.

Gene ontology (GO) analysis examines a representative set of contigs in particular metabolic networks. We examined GO across Day 3 and Day 8 libraries to obtain a general overview of physiological processes predominating at each time point. The GO classification considers 3 categories: molecular function, component (cellular localization), and biological process. To assess the enrichment of molecular function and biological process categories between Day 3 and Day 8 ticks, we examined GO term annotations associated with the encoded contigs. The most substantial enrichments (p < 0.001, n =≥ 5, enrichment ≥1.5) in function and process are shown in Fig. 6. These include enrichments in the molecular functions of chitin binding, cysteine-type endopeptidase, NADH dehydrogenase (ubiquinone), low-density lipoprotein receptor, and lipase activities in the tick gut at day 3 (Fig. 6A), while serine-type endopeptidase inhibitor, methyltransferase, acetylcholinesterase, glutathione transferase, iron binding, monooxygenase, and receptor activities were found to be enriched in the tick gut at day 8 (Fig. 6E). Several enrichments were detected in biological processes, out of which iron transport and catabolic processes of carbohydrates, lipids, and proteins stand out in the tick gut at day 3 (Fig. 6B), while negative regulation of peptidase activity, response to oxidative stress, and xenobiotic metabolism were enriched in the tick gut at day 8 (Fig. 6F). When absolute representation in molecular functions was considered, cysteine-type endopeptidase, glutathione transferase, and glutathione peroxidase activities predominated in libraries of Day 3 ticks (B1‒B4 and S1‒S4), while glutathione peroxidase activity, flavin adenine dinucleotide binding, and serine-type endopeptidase inhibitor activity prevailed in libraries of Day 8 ticks (B5‒B8 and S5‒S8; Fig. 6C,G, respectively). When absolute representation in biological processes was considered, DNA-templated regulation of transcription, protein catabolic processes, and glutathione metabolic processes were over-represented in libraries of Day 3 ticks (B1‒B4 and S1‒S4), while DNA-templated regulation of transcription, negative regulation of peptidase activity, and neuron projection morphogenesis predominated in libraries of Day 8 ticks (B5‒B8 and S5‒S8; Fig. 6D,F, respectively). Although the supplementation of tick diets with gentamicin, necessary to prevent their bacterial decay, may affect expression of some genes responsive to the presence of microbial population in the tick gut, the presence of the antibiotics throughout the feeding in both blood and serum should not substantially misrepresent the obtained data for such comparisons.

RNA-seq analyses have recently enabled exhaustive descriptions of steady-state transcriptome compositions with a remarkable dynamic range between the most abundant and lowly expressed genes, ranging over more than five orders of magnitude. The RNA transcript level of a gene is determined by its regulation at multiple levels, including transcriptional initiation, elongation, splicing, export and degradation^27^. An early belief that there is little correlation between mRNA and protein abundance was recently challenged and, depending on the biological system, mRNA levels were shown to explain up to 90% of changes in protein levels^28^. It can, therefore, be assumed that the transcriptome is a strong predictor of the proteome^29^. Nevertheless, the hierarchical regulation that affects metabolic fluxes in the central metabolism of eukaryotes was reported to be predominantly influenced by post-transcriptional regulation^30^. Therefore, the data collected by GO analysis should serve as a guide that needs to be complemented with protein/metabolite level studies to fully infer physiological processes in the tick gut. Below, we briefly discuss selected categories to gain a better insight into individual functions and processes in tick physiology.

#### Chitin metabolism

Blood-feeding arthropods synthesise a physical barrier along the intestinal lining, called a peritrophic matrix^31^,^32^, which protects intestinal epithelial cells from a blood meal and tick-borne pathogens. Chitin, a linear homopolymer of N-acetyl-β-D-glucosamine, is an important component of such a matrix. We presume that chitin is synthesised endogenously in the tick midgut through a pathway that is conserved throughout lower eukaryotes such as fungi, arthropods, nematodes, or protists^33^. Mapping the chitin biosynthetic pathway in the tick midgut (RPKM ≥3, E value ≤1e-15, and coverage ≥50%; criteria used all the way through the manuscript for mapping and identification of transcripts), we found a single orthologue of glucosamine-phosphate N-acetyltransferase (Ir-114042), UDP-N-acetylglucosamine pyrophosphorylase (Ir-117513), and chitin synthase (Ir-108417). As chitin biosynthesis is absent in vertebrates, chitin biosynthesis and maintenance of chitin integrity is an attractive target not only against fungi^34^, but also against blood-feeding arthropods in order to interfere with their feeding and/or compromise their vectorial capacity^32^.

#### Lipid metabolism

Blood meal plasma contains an abundant lipid fraction of heterogeneous structures including sterols, glycerolphospholipids, glycerolipids, or sphingolipids^35^. Some lipidic compounds are found in higher concentrations in a blood meal cellular fraction, as is the case of sphyngomyosins in erythrocyte membranes^36^. We have noted that lipid catabolism is enriched on day 3 of feeding. There are several copies of phospholipase A2 in the genome of *I. scapularis*^18^. Here, we identified a secreted form of phospholipase A2 (Ir-111829) that is strictly regulated in a temporal-dependent and tissue-specific manner with the Day 3 gut being its prime site of expression. Phospholipase A2 activity was already detected in the secreted saliva of Ixodid ticks^37^. The authors also showed that phospholipase A2 caused haemolysis of sheep red blood cells in an *in vitro* assay and speculated that the enzyme could participate in tick digestive processes *in vivo*^38^.

The low density lipoprotein (LDL) receptor binds LDL, a rich source of host cholesterol, and transports it into cells by endocytosis. Unlike vertebrates, arthropods are incapable of cholesterol biosynthesis *de novo* and must, therefore, acquire it from their diet. In arthropods, exogenous cholesterol is required for endogenous ecdysteroid biosynthesis^5^. Ecdysteroids play a fundamental role in both post-engorgement salivary gland degeneration^39^ and vitellogenesis^40^; for a review, please see^41^. We have found two contigs coding for putative LDL receptor-related proteins: IrSigP-98036 and Ir-112187 that most likely belong to one gene transcript that is orthologous to the *I. scapularis* gene ISCW021767 [VectorBase]. Upon digestion of host lipoproteins, cholesterol is likely bound to tick endogenous lipoproteins and dispatched into the haemolymph for delivery to peripheral tissues. Tick carrier protein, named HeLp, was shown to bind both cholesterol and cholesterol esters in the haemolymph of the tick *Rhipicephalus microplus*^42^. We have identified its putative orthologue (Ir-108832) that is enriched in the gut transcriptomes of Day 8 ticks. Also, cholesterol transport protein and mitochondrial cholesterol transporter were found (IrSigP-116437 and IrSigP-86065, respectively). These proteins likely participate in cholesterol transportation and maintenance of cholesterol homeostasis. The levels of two contigs coding for a putative triglyceride lipase/cholesterol esterase (Ir-107663 and IrSigP-103478) were found to be increased on day 3 of feeding. Interestingly, we have also identified a contig encoding 7-dehydrocholesterol reductase (Ir-120421), the enzyme catalysing the final step of cholesterol biosynthesis.

#### Protein hydrolysis

**Figure 7:**
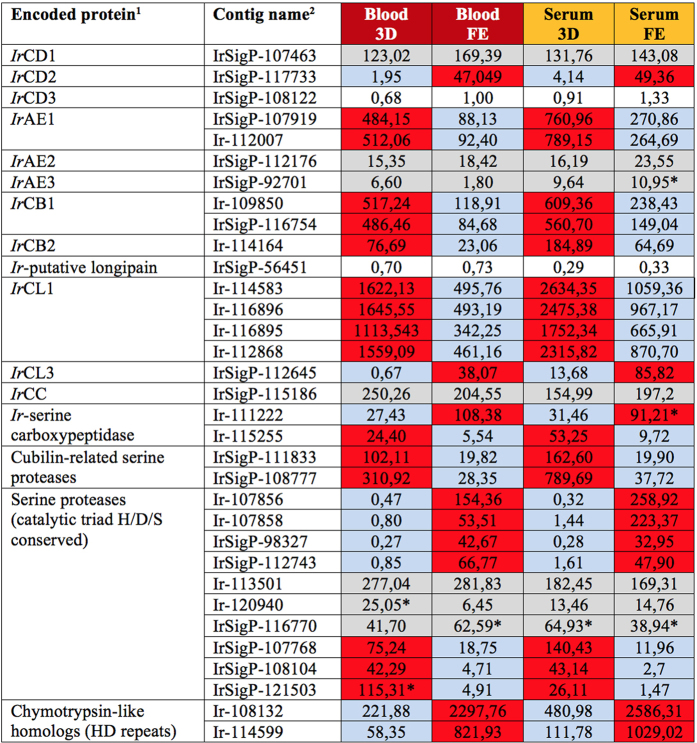
Overview of average RKPM of putative digestive peptidases. Encoded proteins and their respective average RPKM values in particular libraries are listed. ^1^Abbreviations and GenBank Accession Nos: *Ir*CD–*I. ricinus* cathepsin D*, Ir*CD1 (EF428204); *Ir*CD2 (HQ615697), *Ir*CD3 (HQ615698); *Ir*AE–*I. ricinus* asparaginyl endopeptidase (legumain), *Ir*AE1 (AY584752), *Ir*AE2 (ortholog *of I. scapularis* XM_002402043); *Ir*AE3 (unpublished); *Ir*CB–*I. ricinus* cathepsin B, *Ir*CB1 (EF428206); *Ir*CB2 (unpublished); *Ir*-putative longipain (orthologue of *I. scapularis* XM_002433755); *Ir*CL–*I. ricinus* catheppsin L, *Ir*CL1 (EF428205); *Ir*CL3 (ortholog of *I. scapularis* XM_002405329 ); *Ir*CC–*I. ricinus* cathepsin C (EU128750); ^2^For contig names, sequences and other and RKPM data, see the Source data 1 at http://exon.niaid.nih.gov/transcriptome/Ixric-MG/Ir-web.xlsx. Background color coding: No colour-low expression; Grey colour-insignificant differences; Red vs. blue-substantial differences. *-non homogenous expression over four biological replicates.

Ticks and other blood-feeding organisms have evolved an array of lysosomal cysteine proteases (reviewed in ref. 13) that intracellularly digest the protein-rich blood diet within their endo-lysosomal system. Here, we have found contigs coding for members of a papain-type cysteine-protease and asparaginyl-endopeptidase (legumain) families to be among the most abundantly expressed contigs (Source data 1, Column GA‒GP). This finding underscores a critical involvement of protein digestion in tick physiology. One of the principal questions we asked was whether the presence/absence of haemoglobin in the tick diet would affect the levels of transcripts encoding digestive enzymes in the tick gut since serum proteins represent roughly only a third of the total blood protein content. The intracellular digestive apparatus of ticks is localised within the digest cell of the midgut epithelium (Fig. 2) and consists of a network of acidic aspartic peptidases of cathepsin D-type, cysteine exo- and endo-peptidases of cathepsin B, L and C-types (papains), and asparaginyl endopeptidases (AE) of legumain-type^13^,^43^,^44^. The contigs assigned to the *I. ricinus* digestive enzyme isoforms were identified and selected from the Source data 1 and displayed in Fig. 7. RPKM values suggest that there is no apparent difference in levels of digestive peptidase-encoding transcripts between BF and SF ticks, thereby suggesting that their expression is apparently independent of haemoglobin. These results confirm our previous data obtained by qRT-PCR dynamic expression profiling of *I. ricinus* females differentially fed on reconstituted blood or serum^45^. The only exception was reported for *Ir*CB1 that was expressed significantly more in BF ticks on the 5^th^ day of feeding^45^; this could not be verified in the present study, which compared only midgut transcriptomes from *I. ricinus* females fed for 3 and 8 days (fully engorged). Figure 7 clearly indicates that apart from the digestive peptidases up-regulated during the early stages of feeding, there exists another set of “late” peptidase isoforms (*Ir*CD2 and *Ir*CL3) that most likely play a role in blood digestion upon the tick’s drop-off from the host^46^,^47^. Expression of some peptidase isoforms such as *Ir*CD3^46^ or putative longipain^48^ in the *I. ricinus* midgut was negligible, suggesting that these enzymes are not involved in blood-meal digestion. Additionally, we have identified a contig of an as yet undescribed tick cathepsin F (Ir-119743). Whether or not this enzyme is involved in blood meal processing remains to be examined.

Besides the most abundant cysteine and aspartic proteases, contigs encoding two types of serine proteases involved in the tick digestive system^13^,^43^ were also identified: (i) Serine carboxypeptidases were reported to function in the tick as C-terminal monopeptidases, liberating amino-acids from haemoglobin-derived fragments^43^. The serine carboxypeptidase *Hl*SCP1 from *Haemaphysalis longicornis* was characterized and reported to be up-regulated during the course of blood feeding^13^,^49^. The *I. ricinus* orthologue encoded by contig Ir-111222 was also up-regulated in FE ticks while the other isoenzyme encoded by contig Ir-115255 seems to be expressed in the early feeding stage. (ii) Cubilin-related serine peptidase, composed of an N-terminal CUB domain followed by a low-density receptor domain and C-terminal catalytic active serine protease domains were described in *H. longicornis*^50^. This protein is secreted into the gut lumen where it reportedly functions in haemolysis of RBCs in the initial stage of blood-meal processing^13^,^51^. In line with its proposed function, two *I. ricinus* sequences related to cubilin-related serine peptidases were up-regulated during the early feeding stage (Fig. 7).

The *I. ricinus* midgut transcriptomes contain a number of other genes encoding serine proteases with conserved catalytical triad H/D/S. Most of the genes encoding serine proteases were markedly up-regulated in the late stage of feeding, whereas some displayed stable expression or slightly increased expression during the early feeding stage (Fig. 7). Two genes encoding trypsin-like serine proteases, tagged as *Hl*SP2 and *Hl*SP3, with a proposed function in blood digestion, were described in *H. longicornis*. Expression of these genes increased during feeding and the recombinant enzymes had a mildly acidic pH optimum, ~5.0, corresponding to their presumed function in the digest cells and/or midgut lumen^48^. By contrast, our previous results based on selective peptidase inhibitors suggest that serine proteases do not substantially contribute to haemoglobinolytic activity in midgut homogenates from semi-engorged *I. ricinus* females^43^. Therefore, it is possible that the serine proteases expressed towards the end of feeding are active during the off-host stage of blood digestion or have some other, as yet unknown function. Other contigs (Ir-108132 and Ir-114599) that are markedly expressed in *I. ricinus* midgut and up-regulated during feeding, code for non-active chymotrypsin-C homologs having the catalytic triad H/D/S replaced by the residues K/D/L. Corresponding orthologues are present in the *I. scapularis* genome (genes ISCW004734 and ISCW015350, respectively). Earlier, we cloned a related gene from the soft tick *Ornithodoros moubata* (GenBank AAQ82934). An interesting feature of these proteins is the presence of a histidine/aspartic acid-rich motif in the central region of the molecule. A similar motif has been found in hebraein, an 11 kDa antimicrobial protein from the hard tick, *Amblyomma hebraeum* (Lai *et al*., 2004). Whether or not the non-active chymotrypsin homologs play a role in antimicrobial defense within the tick awaits further investigation.

Even though protein hydrolysis yields amino acids essential for vitellogenesis, it also serves as a carbon source for other pathways^52^. During the latter processes, amino acids are deaminated and excessive amino acid-derived nitrogen is disposed of. Most terrestrial invertebrates excrete uric acid or other purines as waste products of nitrogen digestion (uricotelic or purinotelic organisms). These organisms, including ticks, have utilised a pre-existing pathway of purine biosynthesis for disposal of ammonia ions^53^. In purinotelic spiders, mites, and ticks, guanine is the main product of nitrogen digestion^54^,^55^. Here, we have found contigs encoding a full pathway of purine biosynthesis (Ir-110951, Ir-109322, Ir-111239, Ir-108035, Ir-116366, Ir-110916, Ir-107659, Ir-112139, Ir-108403, Ir-109488 and IrSigP-108704). Even though synthesised purines play important roles as putative excessive nitrogen disposal molecules, synthesised and/or salvaged purines also participate in ribonucleotide synthesis for transcription within digest cells, tetrahydrofolate biosynthesis, or as a pheromone signal upon excretion^55^.

#### Negative regulation of proteolytic activity

**Figure 8:**
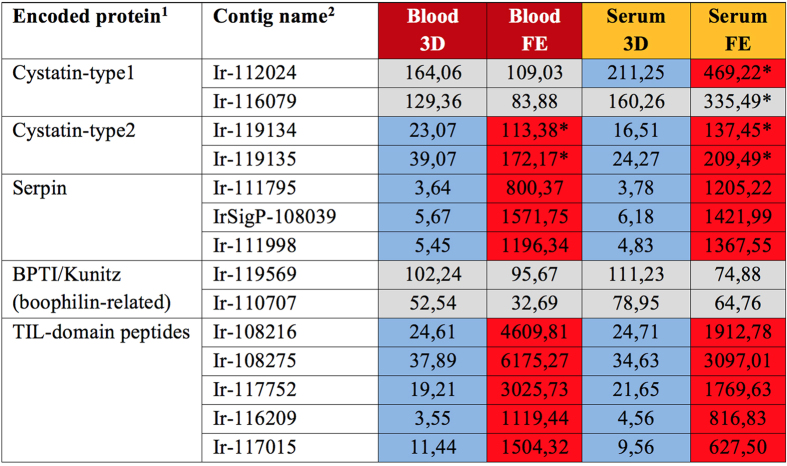
Overview of average RKPM of selected protease inhibitors. Encoded proteins and their respective average RPKM values in particular libraries are listed. ^1^Abbreviations: BPTI-bovine pancreatic trypsin inhibitor, TIL-trypsin inhibitor-like domain (cysteine rich); ^2^contig names, sequences and other and RKPM data, see the Source data 1 at http://exon.niaid.nih.gov/transcriptome/Ixric-MG/Ir-web.xlsx. Background color coding: Grey colour-insignificant differences; Red vs. blue-substantial differences. *-non homogenous expression over four biological replicates.

Contig levels encoding secreted protease inhibitors were substantially enriched in Day 8 ticks. It was recently reported that the midgut transcriptome of *I. ricinus* contains a number of highly expressed protease inhibitors of different classes, including cystatins, serpins, or peptides containing trypsin inhibitor-like (TIL) or Kunitz domains^7^. As the previous study was focused on transcriptomes from early stage feeding (up to 36 hours in adult ticks), roughly corresponding to our Day 3 libraries, our data has allowed disclosure of a further remarkable increase in expression of the majority of putative protease inhibitors, as most of them were up-regulated at full engorgement (Fig. 8).

The role of midgut cystatins is likely associated with the regulation of digestive cysteine peptidases and is presumed to prevent their undesired proteolytic activity within the mature digest cells of the midgut epithelium^3^,^13^,^56^. We noted a marked up-regulation of serpins and putative TIL-domain protease inhibitors in fully engorged BF and SF ticks. We and others have also noted a number of midgut-specific contigs annotated as ‘Kunitz-type’ peptides identified in the *I. ricinus* midgut transcriptome^7^. However, based on a “conserved domain database” search, their sequences do not unequivocally encode an unambiguous Kunitz domain. The genes represented by contigs Ir-119569 and Ir-110707 each contain two BPTI/Kunitz domains and are related to *boophilin*, a Kunitz-type inhibitor from the midgut of the cattle tick *R. microplus*^57^. It was recently suggested that boophilin inhibits proteolytic enzymes of host origin and thereby controls undesired blood coagulation or complement activation within the tick gut lumen^58^. We assume that the tick midgut serine protease inhibitors that are strikingly up-regulated in fully engorged females may function against exogenous serine proteases, either to control haemostasis or inhibit premature digestion of blood-meal stored in the midgut lumen.

#### Response to oxidative stress (redox homeostasis, antioxidant defence, and detoxification)

**Figure 9:**
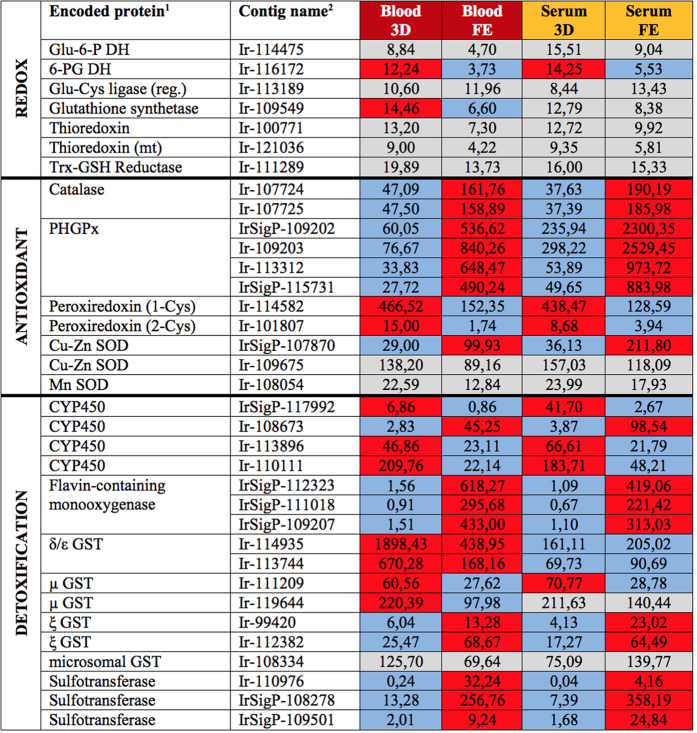
Overview of average RKPM values for contigs encoding redox, antioxidant, and detoxification proteins. Encoded proteins and their respective average RPKM values in particular libraries are listed. ^1^Abbreviations: Glu-6-P DH-Glucose-6-phosphate dehydrogenase; 6-PG DH-6-phosphogluconate dehydrogenase; Glu-Cys ligase-Glutamate-cysteine ligase; Trx-GSH Reductase-Thioredoxin Glutathione Reductase; PHGPx-Phospholipid hydroperoxide glutathione peroxidase; SOD-Superoxide dismutase; CYP450-Cytochrome P450; GST-Glutathione S-transferase; ^2^For contig names, sequences and other and RKPM data, see the Source data 1 at http://exon.niaid.nih.gov/transcriptome/Ixric-MG/Ir-web.xlsx. Background colour coding: Grey colour-insignificant differences; Red vs. blue-substantial differences.

In addition to the maintenance of endogenous redox homeostasis, ticks must cope with the oxidative challenges caused by the host immune system and digestion of a pro-oxidative haemoglobin-containing diet. We have summarised contigs encoding proteins likely participating in redox homeostasis, antioxidant defence, and detoxification in Fig. 9. The glutathione and thioredoxin pools are responsible for the activity of antioxidant enzymes in respective cellular compartments. Glutathione is synthesised in two enzymatic steps, catalysed by dimeric glutamate-cysteine ligase (regulatory and catalytic subunit) and glutathione synthetase. In our midgut libraries of adult *I. ricinus* females, we could find contigs encoding glutathione synthetase (Ir-109549) and a regulatory subunit of glutamate-cysteine ligase (Ir-109549) but we failed to identify the catalytic subunit of this enzyme that was, in contrast, present in the salivary gland transcriptome (BioProject:PRJNA 177622). Unlike glutathione, thioredoxin is a thiol-containing peptide encoded by a single gene. Here, we have identified contigs encoding both cytosolic and mitochondrial putative thioredoxins (Ir-100771 and Ir-121036, respectively), both of which seem to be evenly expressed in all libraries. Both thiols (glutathione and thioredoxin) are recycled by respective reductases at the expense of NADPH. Yeast cells code for two sets of reductases, glutathione reductase and thioredoxin reductase, with affinities to distinct thiols in order to restore their reduced state^59^. Platyhelminth parasites, however, were reported to code for an enzyme glutathione-thioredoxin reductase, displaying affinities towards both oxidised thiols in order to restore their reduced forms^60^. Here, we have found a contig (Ir-111289) encoding a putative glutathione-thioredoxin reductase. NADPH is synthesised predominantly in the pentose phosphate pathway by its two enzymes, glucose-6-dehydrogenase and 6-phosphogluconate dehydrogenase. Contigs encoding homologous proteins were identified as Ir-114475 and Ir-116172, respectively (Fig. 9).

Aerobic organisms have evolved an efficient set of enzymes that can deal with the reactive metabolites of oxygen respiration. Catalase, the first-line antioxidant enzyme that ensures the removal of hydrogen peroxide molecules, is encoded by a single gene in the *I. scapularis* genome [VectorBase; ISCW017124]. Therefore two identically expressed contigs identified in our libraries (Ir-107724 and Ir-107725), likely encode one protein product. Despite the fact that catalase is a haem-containing enzyme, we did not observe any expressional difference of the contigs in relation to dietary RBCs (+/) (Fig. 9). The second group of hydrogen peroxide-removing enzymes is represented by non-haem peroxidases. They are divided into two major subfamilies: (i) glutathione peroxidases (GPx) and (ii) thioredoxin peroxidases (peroxiredoxins). Our data show that contigs (IrSigP-109202, Ir-109203, Ir-113312 and IrSigP-115731) encoding putative *I. ricinus* phospholipid-hydroperoxide glutathione peroxidases (PHGPx), together with catalase, were significantly up-regulated in Day 8 ticks (Fig. 9). The PHGPx class enzymes specifically protect the cell by reducing hydroperoxides of phospholipids, thereby preventing membrane lipoperoxidation. They were also suggested to be involved in resistance to acaricides in the tick *R. microplus*^61^. Our results suggest that the reduction of hydrogen peroxide and phospholipid peroxides is more important during the rapid engorgement and/or off-host digestive phases. Peroxiredoxins contain one or two reactive cysteines (1-Cys or 2-Cys) in their active sites. Here, we have identified one contig (Ir-101807) encoding 2-Cys peroxiredoxin and one highly expressed contig (Ir-114582) encoding a protein with homology to 1-Cys peroxiredoxin. The latter is an orthologue of *I. scapularis* immuno-dominant salivary gland antigen, tagged as Salp25D^62^. This protein, which exerts glutathione peroxidase activity, has been reported to facilitate the acquisition of *Borrelia burgdorferi* spirochetes by the tick^62^,^63^. Superoxide dismutase (SOD) participates in cellular antioxidant defence as it converts highly reactive superoxide radicals to peroxides^64^. SODs are metallo-enzymes that contain Cu, Zn, or Mn in their cores. The *I. ricinus* midgut transcriptomes analysed here contain two contigs encoding Cu-Zn SOD (IrSigP-107870 and Ir-109675) and one contig encoding a putative mitochondrial Mn SOD (Ir-108054) out of which only the former seems to be more highly expressed in Day 8 ticks.

The detoxification of xenobiotics, takes place in two phases: (phase I)-biotransformation of xenobiotics via insertion or uncovering of reactive hydrophilic moieties in their structures via oxidation, reduction, or hydrolysis; (phase II)-xenobiotics or their metabolites undergo conjugation reactions with endogenous compounds, e.g. glutathione or saccharides that enhance their excretion^65^. Cytochromes P450 (CYPs), member of the phase I detoxification system form a large family of membrane proteins. Genes encoding CYPs have apparently undergone several multiplications resulting in a several hundreds of *cyp* genes being present in the genome of *I. scapularis*^18^. Here, we have found four moderately expressed contigs (IrSigP-117992, Ir-108673, Ir-113896 and Ir-110111) encoding CYPs. Although CYPs represent another group of proteins that needs haem as a prosthetic group, no apparent relationship between their expression and dietary RBCs (+/−) was noted. Another phase I detoxification enzyme is flavin-containing monooxygenase (FMO), here encoded as one protein product by contigs IrSigP-112323, IrSigP-111018 and IrSigP-109207 (Fig. 9). In contrast to CYPs, FMO utilizes flavin adenine dinucleotide as a prosthetic group and is markedly up-regulated in fully engorged ticks whereas its expression in the early stage of feeding is marginal.

A family of glutathione S-transferases (GSTs) comprise several classes of enzymes, some of which participate in the phase II detoxification system. GSTs catalyse conjugation of reduced glutathione to an array of xenobiotic substrates. Two GST contigs (Ir-114935 and Ir-113744, likely encoding one protein product), share homology with the δ and ε class of GSTs. As discussed above, this particular GST showed quite rare but obvious up-regulation in the presence of dietary haemoglobin (see also Fig. 4). By contrast, no apparent diet-dependent expression was noted for two other contigs (Ir-111209 and Ir-119644) encoding μ class GSTs, two contigs (Ir-99420 and Ir-112382) encoding ξ class GSTs, and one contig (Ir-108334) encoding microsomal GST (belonging to a MAPEG family of proteins). Sulfotransferases (SULT) catalyse the conjugation of sulfuryl groups to a broad array of substrates, often small insoluble molecules, in order to increase their water solubility and decrease their biological activity. Three contigs (Ir-110976, IrSigP-108278 and IrSigP-109501) encode three distinct SULTs out of which the former transcript seemed to be up-regulated in the presence of haemoglobin (see above and the Fig. 4).

#### Iron binding and haem homeostasis

**Figure 10:**
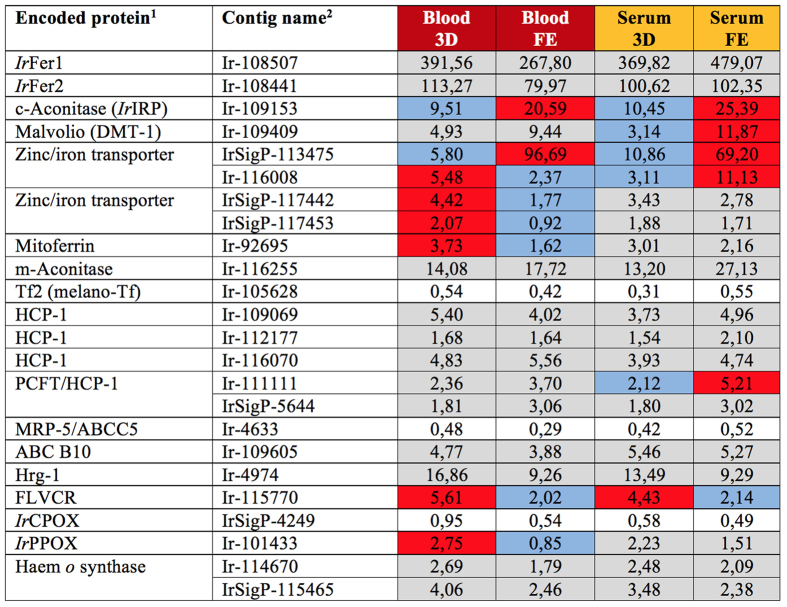
Overview of average RKPM values for contigs encoding proteins involved in iron and haem meatbolism. Encoded proteins and their respective average RPKM values in particular libraries are listed. ^1^Abbreviations and GenBank Accession Nos: *Ir*Fer1-*I. ricinus* ferritin 1 (AF068224); *Ir*Fer2-*I. ricinus* ferritin 2 (EU885951), *Ir*IRP-*I. ricinus* iron regulatory protein/cytoplasmic aconitase (EU885952); DMT-divalent metal transporter; m-Aconitase-mitochondrial aconitase; Tf-transferrin; HCP-haem carrier protein; PCFT-proton-coupled folate transporter; MRP-multidrug resistance protein; Hrg-haem responsive gene; FLVCR-group C feline leukemia virus receptor; *Ir*CPOX-*I. ricinus* coproporphyrinogen oxidase; *Ir*PPOX-*I. ricinus* protoporphyrinogen oxidase; ^2^For contig names, sequences and other and RKPM data, see the Source data 1 at http://exon.niaid.nih.gov/transcriptome/Ixric-MG/Ir-web.xlsx. Background color coding: No colour-low expression; Grey colour-insignificant differences; Red vs. blue-substantial differences.

We recently demonstrated that ticks do not acquire bioavailable iron from haemoglobin, but most likely from host transferrin (Tf)^11^. It is therefore, not surprising that transcripts encoding iron metabolism proteins are not transcriptionally regulated by the presence of dietary haemoglobin (Fig. 10). Mammals internalise Tf through a well described Tf receptor-mediated pathway. Similarly with predictions in insects^66^, we could not find any sequence related to the transferrin receptor in the *I. scapularis* genome^18^ nor in our transcriptomic database (Source data 1). It is only speculative that ticks acquire host transferrin through non-specific endocytosis, similarly to that suggested for host serum albumin^14^. Upon Tf degradation in lysosomes, iron may be transferred to the cytosol through a divalent metal transporter DMT, also termed Malvolio. A contig (Ir-109409) encodes an nramp domain-containing protein that is homologous to insect Malvolio and mammalian DMT-1 protein, both of which have been implicated in intestinal iron absorption and systemic trafficking^67^,^68^. The *I. ricinus* homologue was recently shown to be expressed throughout tick developmental stages and tissues^69^. This work also revealed that RNA-mediated silencing of tick *dmt-1* had no impact on adult *I. ricinus* engorgement or reproductive success^69^. The tick midgut transcriptome also confirmed our previous results^70^ that transcription of the intracellular “storage” ferritin *Ir*-Fer1 (Ir-108507) and the secreted “transporting” ferritin *Ir*Fer2 (Ir-108441) were independent of the course of feeding and further corroborates our data that tick ferritin expression does not respond to dietary RBCs (+/−). The protein levels of *Ir*Fer1 are regulated at the translational level via binding of the iron-responsive protein (IRP) to the iron-responsive element at the 5′ end of *irfer1* mRNA^71^. IRP is an inactive form of the cytosolic aconitase (Ir-109153), which is produced under conditions of iron deficiency. Expression of cytosolic aconitase seems to be slightly up-regulated in fully engorged ticks, independent of haemoglobin. Conversely, mitochondrial aconitase (Ir-116255) was evenly expressed in all transcriptomes. Mitoferrin, a transporter protein facilitating the import of ferrous iron into the mitochondrial matrix, is encoded by the contig Ir-92695 (Fig. 10). We have also noted low levels of contigs encoding transferrin 2, a homologue of insect transferrin 2 (also tagged melanotransferrin)^72^,^73^. Recent work, however, did not confirm the participation of this protein in iron homeostasis or immune defence in the *I. ricinus* tick^69^.

Several types of haem transporter proteins have been, thus far, described in eukaryotic cells: (i) haem carrier protein 1, (ii) haem responsive genes, (iii) feline leukemia virus subgroup C receptor and (iv) ABC transporters. For a more comprehensive review, please see refs 74 and 75. Haem carrier protein (HCP-1) was first described in mouse intestine as a homologue of bacterial metal tetracycline transporters^76^. Using BLAST analysis of the bacterial metal tetracycline transporter (AAG43220), we have identified three contigs (Ir-109069, Ir-112177 and Ir-116070) encoding homologous transmembrane proteins. Two further contigs were detected as homologous to putative HCP-1 (Ir-111111 and IrSigP-5644). In studies exploiting microarray assays in *C. elegans* worms cultured under elevated/reduced levels of haemin, haem-responsive genes (hrg´s) were discovered^77^,^78^. Both HRG-1 and HRG-4 were shown to be essential for haem homeostasis in *C. elegans* and both endogenous proteins were shown to have affinities towards haemin. While HRG-1 was shown to localize in endosomal-lysosomal organelles, HRG-4 was found to localize to the plasma membrane^78^. Recently, the possible *hrg-1* homologue was detected in *I. ricinus* and its levels were reported to be constantly increasing with tick feeding during each developmental stage, with the ovaries of partially and fully engorged females being the prime site of expression^69^. However, in a present study, *irhrg*-1(Ir-4974) showed stable expression in the tick midgut regardless of the feeding time course or diet (Fig. 10). Even though the putative four transmembrane domains were conserved in *Ir*HRG-1, amino acid side chains histidine-90 or the FARKY sequence at the C-terminal, proposed to interact with haem, were not conserved^78^. Therefore, involvement of *Ir*HRG-1 in haem homeostasis in tick tissues remains speculative. Feline leukemia virus subgroup C receptor 1(FLVCR1) was identified in mammalian cells as a member of a major facilitator family of transmembrane transporters and was shown to function as a haem exporter^79^. We have found a contig (Ir-115770) encoding a putative FLVCR that shares 50% and 48% amino acid identities with human FLVCR1 (AAH48312) and FLVCR2 (AAH19087), respectively. A multidrug resistance protein (MRP-5), which belongs to the MRP/ABCC family, was described in *C. elegans* and was implicated in haem export from the worm intestine^80^. A contig (Ir-4633) encoding a tick MRP-5 homolog has been identified. In addition, we found that contig Ir-109605 encoded a protein homologous to the recently reported ABC transporter (ABC B10) that participates in haem transport and detoxification in digest cells of the tick *R. microplus*^81^. Despite the proposed roles of these molecules in maintenance of haem homeostasis, expression of neither MRP-5 -nor ABC B10-related genes seem to be affected by the presence/absence of haemoglobin in the diet. Recently, we have shown that ticks *I. scapularis* and *I. ricinus* do not code for a complete protein set participating in haem biosynthesis^11^. Here, we have found only contigs encoding two out of three vestigial mitochondrial enzymes of the haem biosynthetic pathway, namely coproporphyrinogen oxidase (CPOX) and protoporphyrinogen oxidase (PPOX), whereas a transcript encoding terminal ferrochelatase was absent from our midgut libraries (Fig. 10). Contigs encoding haem *o* synthase were present in our transcriptome (Fig. 10), indicating that acquired host haem is further endogenously metabolised. However, none of these transcripts encoding proteins involved in haem metabolism seemed to be significantly affected by the course of feeding or RBCs (+/−) diet.

## Conclusion

This work is, to our knowledge, the first report describing the use of artificial tick feeding linked with high-throughput transcriptomic analysis aimed at addressing biologically relevant questions such as the tick response to RBC +/− in the diet. In addition, this study has allowed us to perform an as yet unexplored comparison of gene expression in the midgut of individual adult *I. ricinus* females during their early and late stages of feeding. Although we have found a surprisingly low number of transcripts responsive to the presence/absence of haemoglobin, we have identified a great number of contigs associated with temporal-dependent expression in the tick midgut. We believe that our work may pave the way towards experimental manipulation of tick diets in order to enhance our current knowledge of the tick digestive system and tick metabolic demands, with the potential to discover novel target molecules for efficient anti-tick interventions.

## Methods

### Tick maintenance and natural feeding

Larvae from one egg clutch were fed on guinea pigs, under laboratory conditions, to obtain nymphs. These nymphs were fed identically to obtain a genetically-related cohort of adult *Ixodes ricinus* females. These ticks were kept at 24 °C and 95% humidity under a 15:9-hour day/night regime. Adult *I. ricinus* were also collected in the wild near Ceske Budejovice. All laboratory animals were treated in accordance with the Animal Protection Law of the Czech Republic No. 246/1992 Sb., ethics approval No. 357 095/2012. The study was approved by the Institute of Parasitology, Biology Centre of the Czech Academy of Sciences (CAS) and Central Committee for Animal Welfare, Czech Republic (protocol no. 1/2015).

### Tick artificial membrane feeding

Artificial membrane feeding was performed in feeding units manufactured according to the protocol developed by Kröber and Guerin^10^ and adjusted for serum feeding as previously reported^11^. Briefly, serum was obtained from fresh bovine blood samples by centrifugation at 2 500 × g, for 10 min at 4 °C and then the collected serum was spun again at 10 000 × g, for 10 min at 4 °C. The serum was sterile filtered using a 0.22 μm membrane filter (Merck Millipore). To reconstitute the blood without host white cells (reconstituted blood), the serum was supplemented with red blood cells previously washed three times in sterile PBS (haematocrit 45%). Diets were supplemented with ATP (1 mM) and gentamicin (5 μg/ml) and regularly exchanged at 12 h intervals. Females were allowed to attach in 12 h and all unattached ticks were then removed. Males were added at the beginning of the day 3 to complete mating.

### Scanning electron microscopy

Gut caeca were dissected from BF ticks at day 3 of feeding. The tissue was immediately fixed in 2.5% glutaraldehyde in phosphate-glucose buffer (PGB, 0.1M sodium-phosphate, pH 7.2, 4% w/v glucose) for 4 h at room temperature. Tissues were then rinsed with PGB (3 × 15 min). Post-fixation treatment was performed using 2% OsO_4_ for 2 h at room temperature. After washing in PGB, specimens were dehydrated with a graded series of acetone (30 ‒100%) for 15 min at each step, critical point dried (CPD2 Pelco TM) and gold coated using a Baltec SCD 050 sputter coater. The samples were examined in a JSM 7401- F FE-SEM (JEOL Ltd., Tokyo, Japan) at an acceleration voltage of 3 kV using GB-low mode.

### Tick gut preparation and total RNA extraction

Tick females were forcibly removed from the membrane at specified time-points and individual females were dissected under ice-cold DEPC-treated PBS so that clear gut preparations were obtained for RNA extraction. The purity of dissected midguts was examined and all contaminating tissues were removed. Gut preparations were homogenized in RA1 buffer (Machery Nagel) supplemented with β-mercaptoethanol using a 29G syringe. Total RNA was then extracted using a Nucleospin RNA kit (Machery Nagel).

### Illumina Sequencing

Total RNA samples were submitted to the North Carolina State Genomic Sciences Laboratory (Raleigh, NC, USA) for Illumina RNA library construction and sequencing. The amplified library fragments were purified and checked for quality and final concentration by using an Agilent 2100 Bioanalyser with a High Sensitivity DNA chip (Agilent Technologies, USA). The final quantified libraries were pooled in equimolar amounts for sequencing on one lane, according to the MiSeq protocol, and single libraries on four lanes of an Illumina HiSeq 2500 DNA sequencer, utilising a 125 bp single end sequencing flow cell with a HiSeq Reagent Kit v4 (Illumina, USA). Flow cell cluster generation for the HiSeq2500 was performed using an automated cBot system (Illumina, USA). The Real Time Analysis (RTA), version 1.18.64 software package was used to generate raw bcl, or base call files, which were then de-multiplexed by sample into fastq files for data submission using bcl2fastq2 software version v2.16.0. The raw fastq files were deposited in the Sequence Read Archives (SRA) of the National Center for Biotechnology Information (NCBI) under accession number SRP070144 of Bioproject PRJNA311553 and Biosample SAMN04485566.

### Bioinformatics Analyses

Assembly of all reads was carried out as described previously using the Abyss and Soapdenovo-Trans assemblers with every o kmer ending in 1 and 5 (-k program switch) from 21 to 95^15^. Resulting contigs were re-assembled by a pipeline of blastn and a cap3 assembler. Coding sequences were extracted based on blastx results deriving from several database matches, including a subset of the non-redundant protein database of the NCBI containing tick and other invertebrate sequences, as well as the Swissprot and Gene Ontology (GO) databases. Open reading frames larger than 150 nt were also extracted if they had a signal peptide indicative of secretion, as evaluated by version 3.0 of the SignalP program. Reads from each library were mapped back to the CDS by blastn with a word size of 25 and allowing one gap. Reads were mapped up to a maximum of five different CDS if the blast scores were the same for all matches. The edgeR program was used in ancova mode to detect statistically significant differentially expressed genes according to time or diet variables^82^. EdgeR inputted the read matrix for genes having at least one library expressing an FPKM (fragments per thousand nucleotides per million reads) equal or larger than 10. For heat map display of the CDS temporal expression, Z scores of the FPKM values were used. Heatmaps were produced using gplots and heatmap.2 programs with R^82^. All deduced coding sequences and their reads are available for browsing in hyperlinks to several databases (Source data 1).

### Gene ontology analysis

To identify Gene ontology (GO) terms that are differentially expressed in the set of differentially expressed genes, we normalized the RPKM reads for each transcript by dividing them by the average RPKM of the 16 values, and then submitted the pooled values for each of the 4 experimental groups for Kruskal-Wallis analysis (using the R package). The resulting p values were corrected for multiple testing using the Bonferroni implementation in R. Of the 2,685 differentially expressed genes, 1,506 had a match to a GO functional annotation (E-value ≤1e-15) leading to the characterisation of 1,047 transcripts associated with 312 GO functions that were significantly different among the 4 treatments. A total of 1,521 differentially expressed genes matched GO sequences having a component annotation (E-value ≤1e-15). Of these, 1,266 sequences were significantly associated with 108 GO component terms. Finally, 1,530 transcripts matched GO sequences (E-value ≤1e-15) having a process annotation. Of these, 926 had statistical significance, grouping into 328 processes.

### cDNA synthesis, and reverse transcription-quantitative PCR (RT-qPCR)

cDNA preparations from tissues were made in independent triplicates and served as templates for subsequent quantitative expression analyses by RT-qPCR. Samples were analysed using a LightCycler 480 (Roche) and Fast Start Universal SYBR Green Master Kit (Roche). Each primer pair (for the list of qPCR primers, see Supplementary Table S3) was inspected for its specificity using melting curve analysis. Relative expressions were calculated using the ΔΔCt method. The expression profiles from adult *I. ricinus* female tick tissues were normalised to *actin* or *ef-1α*.

## Additional Information

**How to cite this article**: Perner, J. *et al*. RNA-seq analyses of the midgut from blood- and serum-fed *Ixodes ricinus* ticks. *Sci. Rep.* **6**, 36695; doi: 10.1038/srep36695 (2016).

## Supplementary Material

Supplementary Information

## Figures and Tables

**Table 1:** Overview of contigs^1^ over-represented in libraries B1‒B4 and S1‒S4 (partially-fed stage) over libraries B5‒B8 and S5‒S8 (fully-engorged stage).

Contig Name	Encoded protein	Fold Change	E value	Coverage (%)	Protein Database
***Ir-111829***	Phospholipase A2	2131	0	109.8	NR-LIGHT
***Ir-103540***	Sphingomyelin phosphodiesterase	669	0	44.6	NR-LIGHT
***Ir-110985***	Acid sphingomyelinase	614	0	66	KOG
***Ir-108861***	Lipase	492	0	98	NR-LIGHT
***IrSigP-110295***	Phosphoenolpyruvate synthase	451	1e-179	81	NR-LIGHT
***IrSigP-109251***	Glycine-rich secreted cement protein	221	0	102	NR-LIGHT
***Ir-109833***	Glycine-rich secreted cement protein	119	0	80.1	SWISSP
***Ir-114115***	Sulfotransferase	94	0	63.6	ACARI
***IrSigP-114509***	Phosphatidylinositol- phospholipase c domain	81	0	100	ACARI
***Ir-98671***	Arginine kinase	68	1e-173	99	SWISSP
***Ir-100072***	Estradiol 17-beta-dehydrogenase 8	50	5e-046	98	SWISSP

^1^RPKM ≥3, E value ≤1e-15, and coverage ≥50%.

**Table 2:** Overview of contigs^1^ over-represented in libraries B5‒B8 and S5‒S8 (fully-engorged stage) over libraries B1‒B4 and S1‒S4 (partially-fed stage).

Contig Name	Encoded protein	Fold Change	E value	Coverage (%)	Protein Database
***IrSigP-107534***	Vesicular amine transporter	634	0	87	NR-LIGHT
***Ir-121498***	Acyl-CoA synthetase	520	0	99	NR-LIGHT
***Ir-113115***	JH acid methyltransferase	441	7e-96	60	ACARI
***Ir-97790***	GSH peroxidase	339	9e-62	97	ACARI
***Ir-113156***	Kunitz 80	325	2e-40	80	ACARI
***IrSigP-108669***	Vitellogenin 2	211	0	102	NR-LIGHT
***Ir-109200***	Organic anion transporter	109	0	87	KOG
***Ir-118795***	Estradiol 17-beta-dehydrogenase 8	99	2e-49	97	SWISSP
***Ir-113345***	15-OH prostaglandin dehydrogenase	91	7e-44	90	KOG
***Ir-113452***	SEC14	85	3e-35	93	KOG
***IrSigP-113423***	Acetylcholinesterase	43	0	91	KOG
***Ir-108832***	Carrier protein 6	26	0	90	NR-LIGHT

^1^RPKM ≥3, E value ≤1e-15, and coverage ≥50%.
