# Supplementary material for: RNA-seq analyses of the midgut from blood- and serum-fed Ixodes ricinus ticks
Source: Sci Rep. 2016 Nov 8;6:36695. doi: 10.1038/srep36695 (PMC5099782; doi:10.1038/srep36695)
Supplement: Supplementary Information [file srep36695-s1.pdf]

Supplementary Information

RNA-seq analyses of the midgut from blood- and serum-fed *Ixodes ricinus* ticks

Jan Perner, Jan Provazník, Jana Schrenková, Veronika Urbanová,

José M. C. Ribeiro, Petr Kopáček

Table S1. The summary of Miseq the reads after removal of Illumina primers and trimming low base quality (smaller than 20).

Library name	Meal	Time in days	Total number of sequences	Total number of residues	Average length	Median size	L50
B1	Blood	3	2,155,570	623,472,674	289.2	300	300
B2	Blood	3	3,413,789	983,125,506	287.9	300	300
B3	Blood	3	1,913,967	547,583,431	286.0	300	300
B4	Blood	3	2,747,297	786,753,771	286.3	300	300
B5	Blood	8	3,203,379	903,713,423	282.1	300	300
B6	Blood	8	2,371,577	677,111,683	285.5	300	300
B7	Blood	8	3,108,306	892,945,075	287.2	300	300
B8	Blood	8	4,172,957	1,188,549,527	284.8	300	300
S1	Serum	3	3,213,133	912,458,003	283.9	300	300
S2	Serum	3	3,118,604	878,902,497	281.8	300	300
S3	Serum	3	3,007,399	840,317,863	279.4	300	300
S4	Serum	3	3,228,533	903,949,950	279.9	300	300
S5	Serum	8	3,064,434	866,807,467	282.8	300	300
S6	Serum	8	2,395,662	670,246,143	279.7	300	300
S7	Serum	8	2,601,754	726,967,206	279.4	300	300
S8	Serum	8	2,507,908	678,757,731	270.6	300	300
**Total**			46,224,269	13,081,661,950			
**Average**			2,889,017	817,603,872	283		
**Minimum**			1,913,967	547,583,431			
**Maximum**			4,172,957	1,188,549,527			

Table S2. The summary of Hiseq the reads after removal of Illumina primers and trimming low base quality (smaller than 20).

Library name	Meal	Feeding time in days	Total number of sequences	Total number of residues	Average length	Median size	L50
B1	Blood	3	9,629,494	1,190,648,679	123.7	125	125
B2	Blood	3	15,544,323	1,860,018,389	119.6	125	125
B3	Blood	3	11,884,809	1,320,412,881	111.1	125	125
B4	Blood	3	12,704,580	1,479,349,109	116.4	125	125
B5	Blood	8	15,348,566	1,766,877,794	115.1	125	125
B6	Blood	8	11,105,251	1,303,445,535	117.3	125	125
B7	Blood	8	14,939,645	1,754,368,386	117.4	125	125
B8	Blood	8	15,951,632	1,878,440,011	117.7	125	125
S1	Serum	3	15,033,927	1,821,330,422	121.1	125	125
S2	Serum	3	13,825,806	1,651,700,131	119.5	125	125
S3	Serum	3	15,959,483	1,902,584,122	119.2	125	125
S4	Serum	3	14,125,114	1,692,400,404	119.8	125	125
S5	Serum	8	11,945,337	1,470,248,650	123.0	125	125
S6	Serum	8	10,181,106	1,255,324,249	123.3	125	125
S7	Serum	8	10,753,913	1,325,093,390	123.2	125	125
S8	Serum	8	7,485,714	917,067,908	122.5	125	125
**Total**			206,418,700	24,589,310,060			
**Average**			12,901,169	1,536,831,879	119		
**Minimum**			7,485,714	917,067,908			
**Maximum**			15,959,483	1,902,584,122			

Table S3: List of oligonucleotides used in this work for RT-qPCR analyses of direct and independent validations, tissue profile expression of Day-3 up-regulated contigs, and tissue profile expression of Day-8 up-regulated contigs

Transcript	Contig no.	Forward primer (5´-3´)	Reverse primer (5´-3´)
gst	Ir-114935	ggttcgtcctgtgggaaag	actgtccggggcgtactt
gst	Ir-113744	ttatgcatttggatgcttcg	gccctgtcaggttggtcata
sult	Ir-110976	ggtcgacagagttccctacg	tcccacgggttacgatagac
ache	Ir-108903	cgtttctcatgagtggcaca	tcggactgcactctcgaac
Na+-bile acid c-t	IrSigP-109984	atggcatcgtggcaactc	gggatggacccgtagctc
ph-gpx	IrSigP-109202	ctggaagtacctcaaggaaagg	ctgtccattcttgtccacca
pm-chbp	Ir-113572	cgacaacagctatcggatga	aggtgtggtagcgacgaatc
cyp450	IrSigP-117992	gcgaaggagcaggttctct	agatttggttcgggtccag
m20	Ir-107651	caacatctccatgcttcgac	cattaagcatcaccccatga
cyp450	IrSigP-112182	ctcaggagaccgctgagttt	gcgttatattgccggaaaag
unknown	IrSigP-112102	gtgctacaaagccaacgaca	ggacggtggaggaaggtt
unknown	Ir-111905	atgagcccattatcctgacg	gacacggtgagagtggtctg
unknown	IrSigP-107775	acgggaatgaacttgacagc	cgacaatgtttcggattctg
unknown	IrSigP-111681	ctgaaaggagtgaccgttgc	ggccgacaccttgttgaa
plA2	Ir-111829	gacaactccgacgacaacatc	agaactggcggtcatctttg
smpd	Ir-103540	gccctcatcaagctgctc	ctcgtccttgtagaggtctatgg
a-smase	Ir-110985	agtcggtccccatcaacag	gccactggacggtgtagttt
lip	Ir-108861	catcttttcgcactccgtct	cgggagtagagcatgaacttg
pep synthase	IrSigP-110295	tcgagtacaaatggcagcac	cacgtcagactgcaccatct
grp	IrSigP-109251	caggaagaaacgggcaagta	ccggaggtccttctggtc
grp	Ir-109833	tggttctaagggcgagaaag	aaacctggtagtccatttcgtc
sult	Ir-114115	cttacgactgttgcgtgtcc	cgaactggtaagcagggaag
plc	IrSigP-114509	cctagtggcgatggatttct	ttagcatcgatggcaacatt
ak	Ir-98671	ggcaacactagacaagctgga	agcgacttgcagtccttagc
hsd17b8	Ir-100072	tcacaagtggatgtccggta	caccacgatgcttaaaggaag
vmat	IrSigP-107534	ggattctacatccgggacac	gcagaacccacgtcactgt
acyl-CoA synthetase	Ir-121498	gttgtactgcgcaactcgtc	aacgaagtccttgagtttctcg
jh-acid mt	Ir-113115	gaaagaaatggtcgcagacg	tggactagcggggtaaacag
gpx	Ir-97790	ccagaagatcgaggtgaacg	aacgacccttgaggaaagtgt
kunitz 80	Ir-113156	atgttccggcgctacaaat	cgcagcctccgtaaatgt
vitellogenin 2	IrSigP-108669	cctcacttccctccagacttc	ccaggagcaagtcgaggac
org. anion trans.	Ir-109200	gaatgttgctgtcggtcttg	tgccgtggattccgtaag
hsd17b8	Ir-118795	gcaagtgtgggtatgccttt	gacttcgtgaacgcaatgac
15-oh-pg-dh	Ir-113345	tcgttgtcaataatgcaggtg	aagcagggttccacagaatg
sec14	Ir-113452	caattgtccggcatctga	tttgttccgtgaaggtggat
ache	IrSigP-113423	gaacctacgacgcaacgact	gtggtttcttcacgttctcaagt
ir-cp6	Ir-108832	catcgaggatcacgagagg	tgatcatcatcttggagtacgg
ef	IrSigP-108204	acgaggctctgacggaag	cacgacgcaactccttcac
actin	Ir-107867	cgacatcaaggagaagctctg	gtcgggaagctcgtaggac

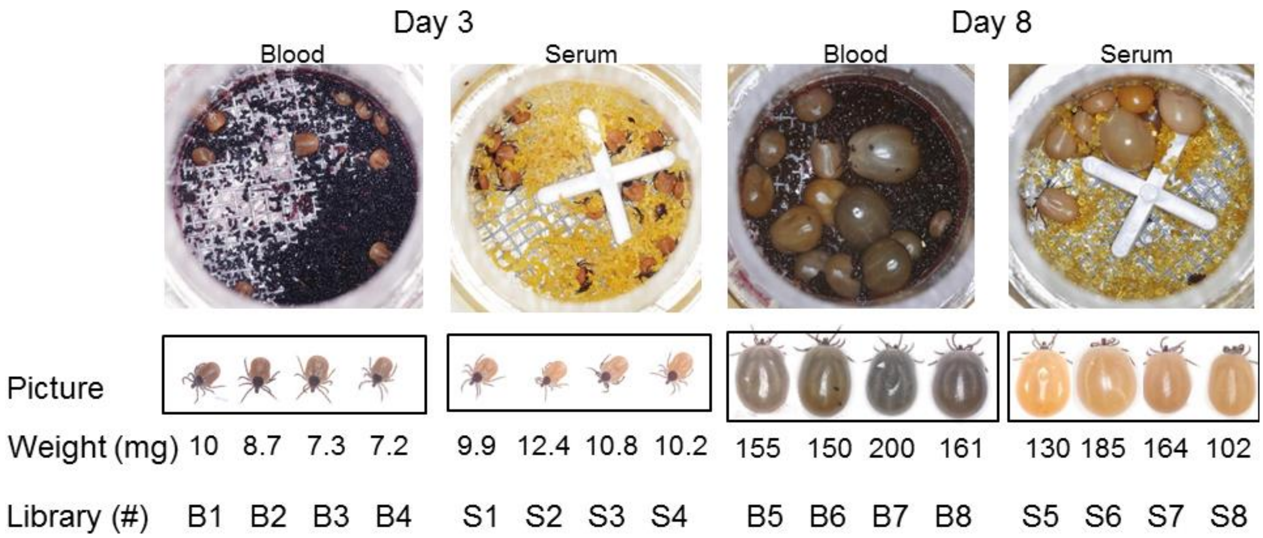

Figure S1. Overview of the experimental design and libraries designation. Libraries were prepared from individual first-generation siblings *Ixodes ricinus* females fed *in vitro* for 3 days or 8 days. Females were fed either reconstituted bovine blood or bovine serum. Similarly appearing tick females were forcibly removed at indicated time points. Weights of individual females and corresponding library names, used in Source data 1, are shown.

A)

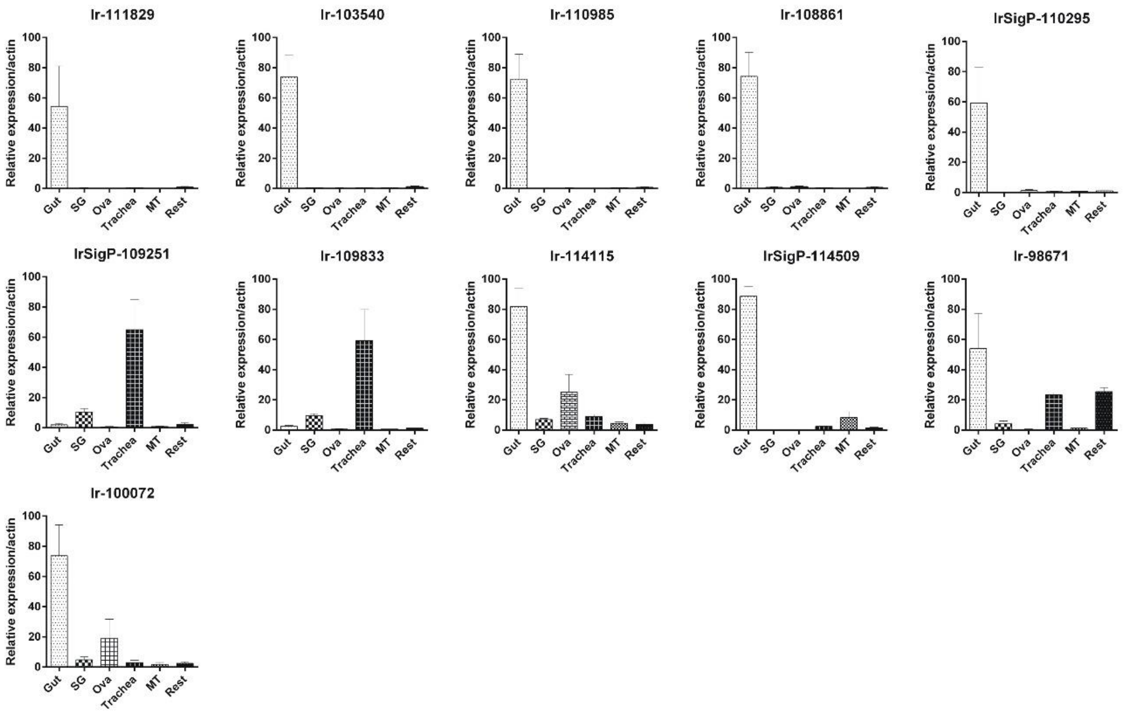
Ir-111829
Ir-103540
Ir-110985
Ir-108861
IrSigP-110295
IrSigP-109251
Ir-109833
Ir-114115
IrSigP-114509
Ir-98671
Ir-100072
Relative expression/actin
Gut
SG
Ova
Trachea
MT
Rest

B)

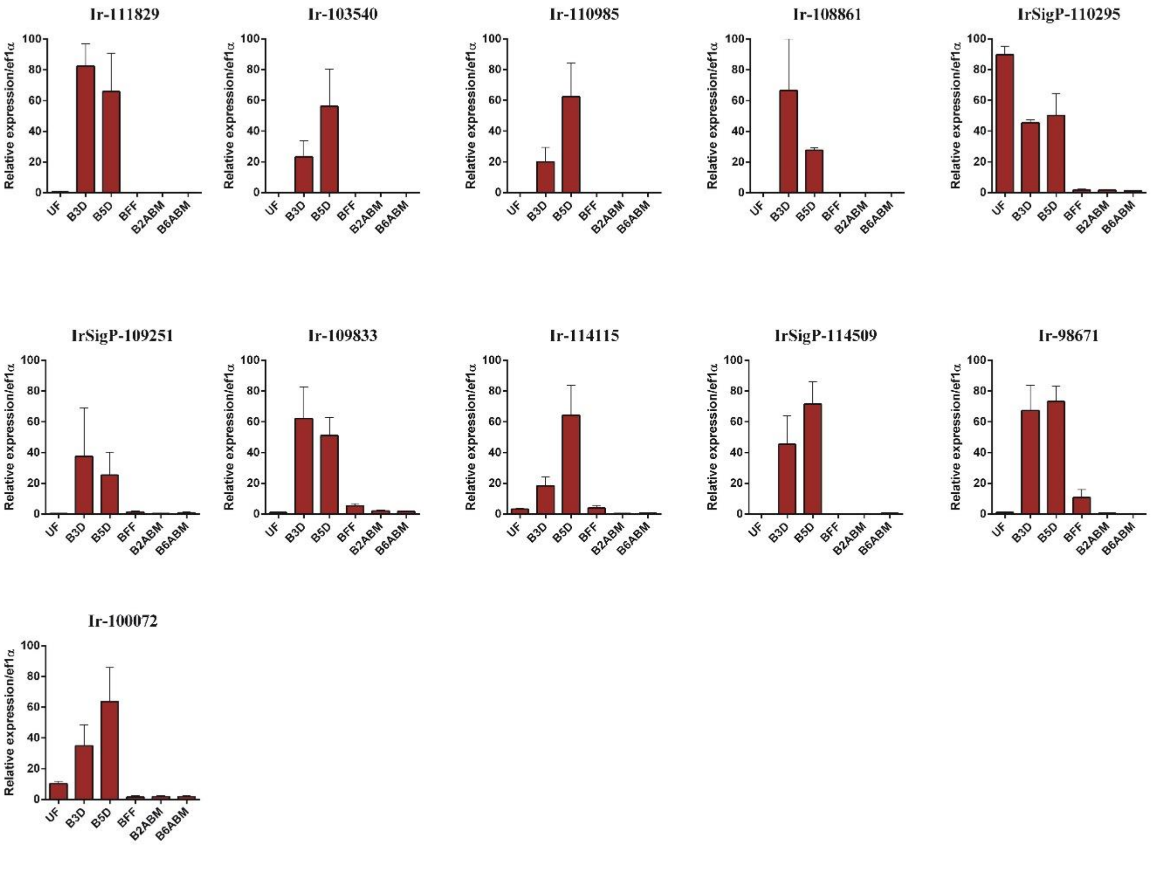
Ir-111829
Ir-103540
Ir-110985
Ir-108861
IrSigP-110295
IrSigP-109251
Ir-109833
Ir-114115
IrSigP-114509
Ir-98671
Ir-100072
Relative expression/ef1α
UF
B3D
B5D
BFF
B2ABM
B6ABM

Figure S2. RT-qPCR profiles of genes over-represented in the gut of 3D ticks. A) cDNA samples were synthesised from RNA isolated from dissected tissues of *I. ricinus* females collected in wild, fed naturally for 3 days. SG - salivary glands, Ova - ovaries; Trachea - trachea-fat body complex; MT - Malpighian tubules; Rest - remaining tissues. Mean values and SEM are shown, n = 3 (biological replicates) **B)** cDNA samples were synthesised from RNA isolated from dissected gut of *I. ricinus* females before, during, and after feeding. UF - unfed, B3D - blood-fed for 3 days; B5D - blood-fed for 5 days; BFF - blood-fed for 9 days (fully fed); B2ABM - 2 days after blood-meal; B6ABM - 6 days after blood-meal. Mean values and SEM are shown, n = 3 (biological replicates).

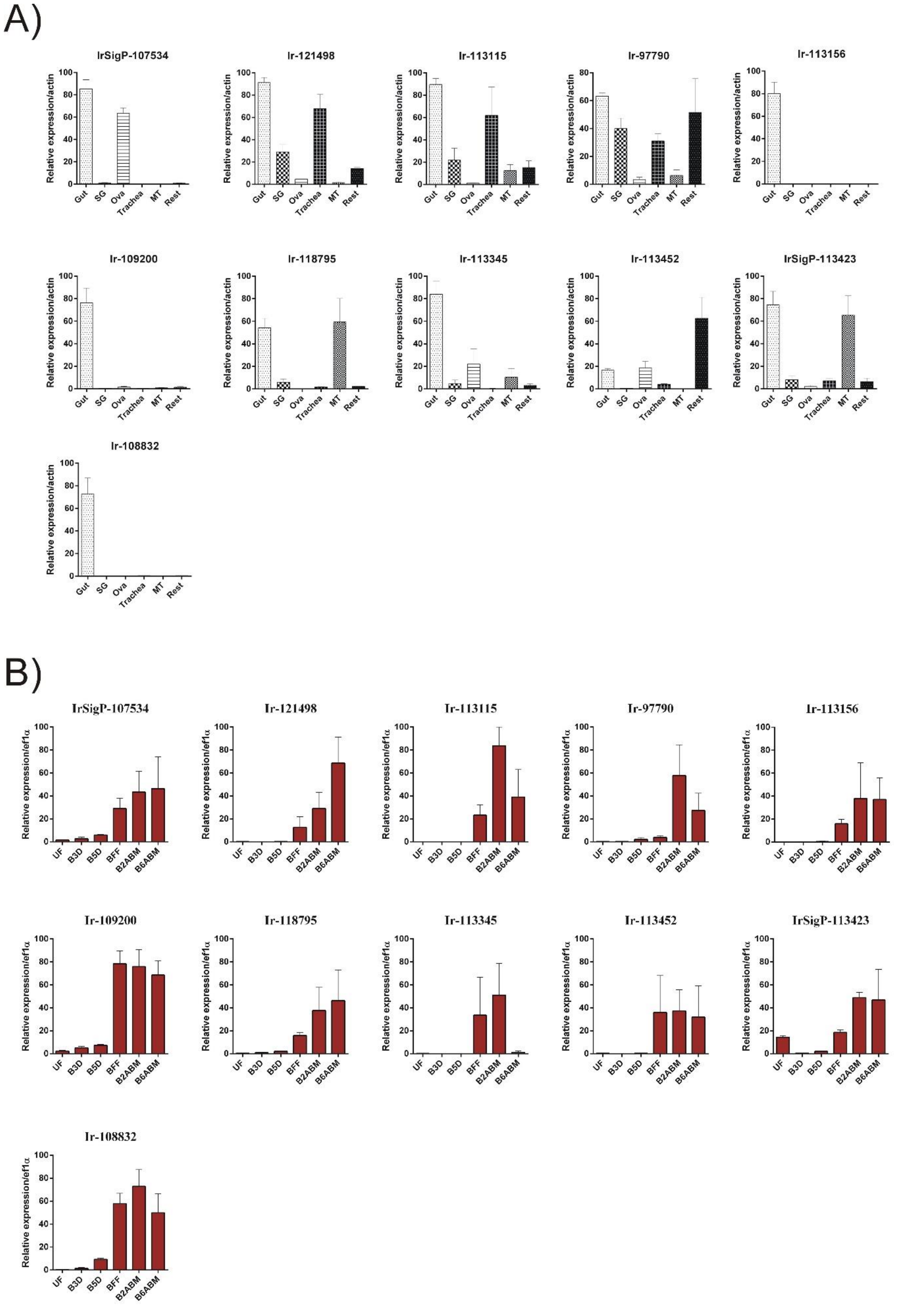
A)
IrSigP-107534
Ir-121498
Ir-113115
Ir-97790
Ir-113156
Ir-109200
Ir-118795
Ir-113345
Ir-113452
IrSigP-113423
Ir-108832
Relative expression/actin
Gut
SG
Ova
Trachea
MT
Rest
B)
IrSigP-107534
Ir-121498
Ir-113115
Ir-97790
Ir-113156
Ir-109200
Ir-118795
Ir-113345
Ir-113452
IrSigP-113423
Ir-108832
Relative expression/ef1α
UF
B3D
B5D
BFF
B2ABM
B6ABM

Figure S3. RT-qPCR profiles of genes over-represented in the gut of 8D ticks. A) cDNA samples were synthesised from RNA isolated from dissected tissues of *I. ricinus* females collected in wild, fed naturally for 3 days. SG - salivary glands, Ova - ovaries; Trachea - trachea-fat body complex; MT - Malpighian tubules; Rest - remaining tissues. Mean values and SEM are shown, n = 3 (biological replicates) **B)** cDNA samples were synthesised from RNA isolates of dissected gut of *I. ricinus* females before, during, and after feeding. UF - unfed, B3D - blood-fed for 3 days; B5D - blood-fed for 5 days; BFF - blood-fed for 9 days (fully fed); B2ABM - 2 days after blood-meal; B6ABM - 6 days after blood-meal. Mean values and SEM are shown, n = 3 (biological replicates).

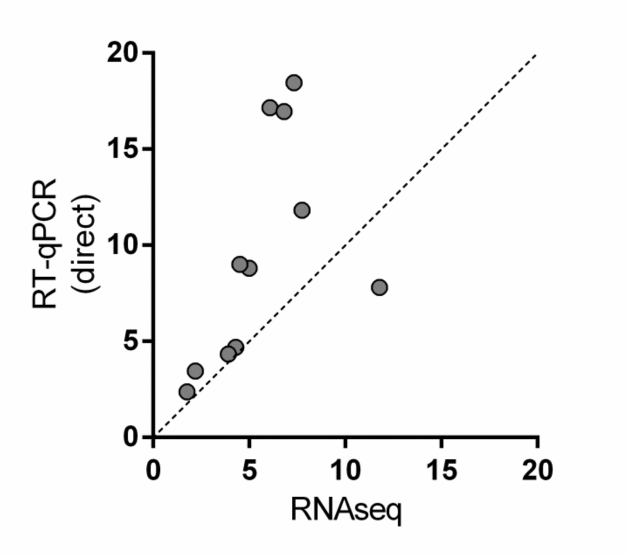

Figure S4. Correlation of direct RT-qPCR validation and RNA-seq data. Individual RT-qPCR fold change up-regulations (based on mean relative expression value from three biological triplicates) are plotted against RPKM up-regulations from RNA-seq data. Following contigs were used as representatives for validation: Ir-114935, Ir-110976, Ir-113572, Ir-108903, IrSigP-117992, IrSigP-109202, Ir-107651, IrSigP-112182, IrSigP-112102, Ir-111905, IrSigP-107775.

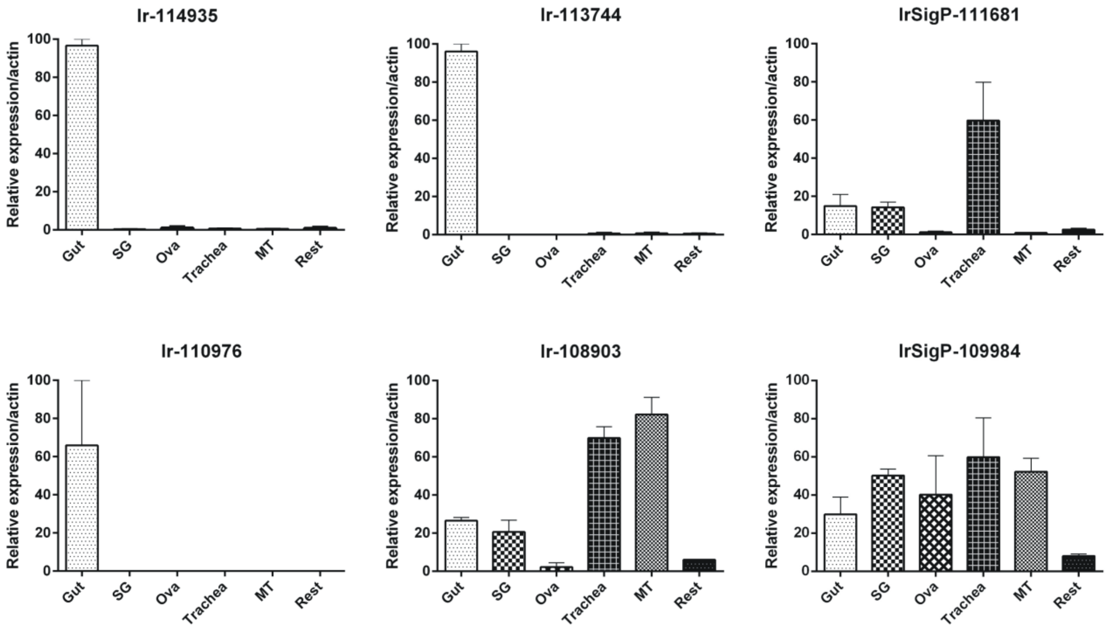

Figure S5. Tissue expression profile of differentially expressed genes in the gut of blood-fed and serum-fed ticks. Three sets of cDNA from selected tissues of naturally fed ticks for 3 days or 8 days were synthesised. Tissue expression profiling was analysed by RT-qPCR at the time-point of differential expression (Day 3 or Day 8). SG - salivary glands, Ova - ovaries; Trachea - trachea-fat body complex; MT - Malpighian tubules; Rest - remaining tissues. Mean values and SEM are shown, n = 3 (biological replicates).
